# Tetra­kis(triphenyl­phosphane-κ*P*)silver(I) trifluoro­methane­sulfonate dichloro­methane monosolvate

**DOI:** 10.1107/S1600536811040670

**Published:** 2011-10-08

**Authors:** Yu-Hang Jiang, Li-Na Cui, Xu Huang, Qiong-Hua Jin, Cun-Lin Zhang

**Affiliations:** a Department of Chemistry, Capital Normal University, Beijing 100048, People’s Republic of China; b Key Laboratory of Terahertz Optoelectronics, Ministry of Education, Department of Physics, Capital Normal University, Beijing 100048, People’s Republic of China

## Abstract

In the title compound, [Ag(C_18_H_15_P)_4_]CF_3_O_3_S·CH_2_Cl_2_, the Ag atom is coordinated by four P atoms from four PPh_3_ ligands. The P—Ag—P angles are in the range 108.02 (6)–110.15 (6)°, which confirms the distorted tetra­hedral environment around the Ag atom.

## Related literature

For background to silver(I)-phosphane-oligodentate N-base complexes in biological processes and luminescence materials, see: Effendy *et al.* (2007 ▶); Jin *et al.* (2010*a* ▶,*b* ▶). For the syntheses of related structures, see: Song *et al.* (2010 ▶); Jin *et al.* (2008 ▶); For related structures, see: Wen *et al.* (2011 ▶); Mu *et al.* (2010 ▶); Cui *et al.* (2010*a* ▶,*b* ▶); Wu *et al.* (2009 ▶).
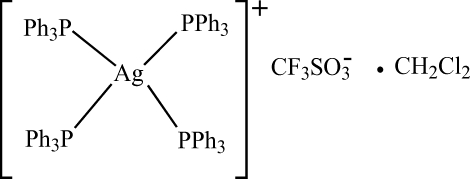


## Experimental

### 

#### Crystal data

[Ag(C_18_H_15_P)_4_]CF_3_O_3_S·CH_2_Cl_2_
*M* *_r_* = 1390.95
Triclinic, 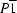

*a* = 11.9868 (11) Å
*b* = 14.7498 (14) Å
*c* = 18.7887 (17) Å
α = 89.613 (2)°
β = 85.858 (1)°
γ = 88.490 (1)°
*V* = 3312.0 (5) Å^3^
*Z* = 2
Mo *K*α radiation
μ = 0.57 mm^−1^
*T* = 298 K
0.45 × 0.44 × 0.24 mm

#### Data collection

Bruker SMART CCD area-detector diffractometer
Absorption correction: multi-scan (*SADABS*; Bruker, 2007 ▶) *T*_min_ = 0.784, *T*_max_ = 0.875
17415 measured reflections
11528 independent reflections
6308 reflections with *I* > 2σ(*I*)
*R*_int_ = 0.045

#### Refinement

*R*[*F* ^2^ > 2σ(*F* ^2^)] = 0.069
*wR*(*F* ^2^) = 0.215
*S* = 1.03
11528 reflections
793 parameters
H-atom parameters constrained
Δρ_max_ = 0.95 e Å^−3^
Δρ_min_ = −0.74 e Å^−3^

### 

Data collection: *SMART* (Bruker, 2007 ▶); cell refinement: *SAINT-Plus* (Bruker, 2007 ▶); data reduction: *SAINT-Plus*; program(s) used to solve structure: *SHELXS97* (Sheldrick, 2008 ▶); program(s) used to refine structure: *SHELXL97* (Sheldrick, 2008 ▶); molecular graphics: *SHELXTL* (Sheldrick, 2008 ▶); software used to prepare material for publication: *SHELXTL*.

## Supplementary Material

Crystal structure: contains datablock(s) global, I. DOI: 10.1107/S1600536811040670/ff2029sup1.cif

Structure factors: contains datablock(s) I. DOI: 10.1107/S1600536811040670/ff2029Isup2.hkl

Additional supplementary materials:  crystallographic information; 3D view; checkCIF report

## Figures and Tables

**Table 1:** Selected bond lengths (Å)

Ag1—P1	2.6202 (17)
Ag1—P3	2.6480 (17)
Ag1—P4	2.6494 (18)
Ag1—P2	2.6682 (17)

**Fig. 1.**
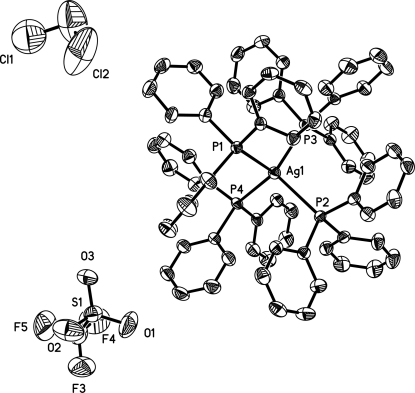
Ellipsoid plot of the title compound. Hydrogen atoms are omitted for clarity. Atoms are displayed as ellipsoids at the 50% probability level.

## supplementary crystallographic information

### Comment

Reports concerned with the structural and kinetic features of silver(I)-phosphane-oligodentate N-base complexes are growing quickly in number after the participation of these compounds in biological process and luminescence materials were discovered (Jin *et al.*, 2010*a*, 2010*b*, Effendy *et al.*, 2007). During the study of complexes of mixed P and N-ligands with special structure, we found that some heterocylic N-ligands act as catalyst in the reaction. For example ,[Ag_4_SCN_4_dppm_2_] and [AgSCNdppm]_2_ were obtained under the catalysis of quinoline and phenathroline(Phen), respectively (Jin *et al.* 2008, Song *et al.*, 2010). [AgClO_4_(PPh_3_)_3_](Cui *et al.*, 2010*a*), [AgClO_4_(PPh_3_)_3_(MeOH)] (Cui *et al.*, 2010*b*) and [Ag(PPh_3_)(CH_3_COO)]_2_.H_2_O.CH_3_OH (Mu *et al.*, 2010)are prepared under the catalysis of 2-aminopyrimidine. Recently, using similar synthesis strategy we obtained the complex [Ag(PPh_3_)_4_](CF_3_O_3_S) (2) (Wen *et al.*, 2011). Herein, we report the complex [Ag(PPh_3_)_4_](CF_3_O_3_S).CH_2_Cl_2 _(1), which is unexpectedly obtained in the experiment trying to prepare an Ag(I) complex of ADMT and PPh_3_.

Though complexes 1 and 2 are very similar, they have different space groups (P-1 for 1 and *R*-3 for 2) and different formulas ([Ag(PPh_3_)_4_](OTf).CH_2_Cl_2_ for 1 and [Ag(PPh_3_)_4_](OTf) for 2. In complex 1, the angles P—Ag—P are in the range of 108.02 (6)–110.15 (6)°, which confirms the distorted tetrahedral environment around the silver atom. The Ag is coordinated by four P atoms from PPh_3_. The four Ag—P distances are in the range of 2.6202 (17)–2.6682 (17) Å, which are longer than those in [AgClO_4_(PPh_3_)_3_] (2.5047–2.564 (1) Å), [AgClO_4_(PPh_3_)_2_(MeOH)] (2.430 (8)–2.4276 (11) Å) and [Ag(PPh_3_)(Phen)]CF_3_SO_3_ (2.29 (2) Å) (Wu *et al.*,2009), but are similar to those in complex 2 (2.624 (2)–2.6538 (12) Å). P—Ag—P angles are in the range of 108.02 (6)–110.15 (6)°, which are smaller than those in [AgClO_4_(PPh_3_)_3_](114.70–119.17°), [Ag(PPh_3_)(CH_3_COO)]_2_.H_2_O.CH_3_OH (128.13–128.27 (6)°) and [AgClO_4_(PPh_3_)_2_(MeOH)] (133.5°),but are similar to those in complex 2 (109.45 (3)–109.50 (3)°).

The new Ag(I)-PPh_3_—OTf complex was synthesized through two-step method. First the adduct of Ag:ADMT (1:4) was got by the reaction of AgOTf with ADMT, then PPh_3_ substituted all four ligands ADMT of Ag:ADMT adduct to get complex 1. Though complex 1 is similar to complex 2, the procedure of preparing complex 1 is clearer, which makes the mechanism involving N-heterocyclic ligands more understandable.

### Experimental

A mixture of AgOTf (silver trifluoromethanesulfonate) and ADMT (ADMT=3-Amino-5,6-dimethyl-1,2,4-triazine) in molar ratio of 1:4 in the mixed solution of CH_3_CN (5 ml), CH_2_Cl_2_(5 ml) and CH_3_OH (2 ml) was stirred for 6 h at room temperature,then filtered. Subsequent slow evaporation of the filtrate resulted in the formation of yellow crystals of the adduct of AgOTf:ADMT(1:4). Then the title compound was obtained by the reaction of the adduct of AgOTf:ADMT(1:4) with PPh_3_(triphenylphosphane) in molar ratio of 1:1 in the mixed solution of CH_3_OH (5 ml)and CH_2_Cl_2_(5 ml) for 4 h at room temperature. After filtration,the filtrate was evaporated slowly at room temperature to yield white crystals. Crystals suitable for single-crystal X-ray diffraction were selected directly from the sample as prepared.

### Refinement

Hydrogen atoms were located in the calculated sites and included in the final refinement in the riding model approximation with displacement parameters set to 1.2×*U*_eq_ of the parent atom.

### Crystal data

**Table:**

[Ag(C_18_H_15_P)_4_]CF_3_O_3_S·CH_2_Cl_2_	*Z* = 2
*M**_r_* = 1390.95	*F*(000) = 1428
Triclinic, *P*1	*D*_x_ = 1.395 Mg m^−^^3^
*a* = 11.9868 (11) Å	Mo *K*α radiation, λ = 0.71073 Å
*b* = 14.7498 (14) Å	Cell parameters from 3454 reflections
*c* = 18.7887 (17) Å	θ = 2.4–20.9°
α = 89.613 (2)°	µ = 0.57 mm^−^^1^
β = 85.858 (1)°	*T* = 298 K
γ = 88.490 (1)°	Prism, white
*V* = 3312.0 (5) Å^3^	0.45 × 0.44 × 0.24 mm

### Data collection

**Table:**

Bruker SMART CCD area-detector diffractometer	11528 independent reflections
Radiation source: fine-focus sealed tube	6308 reflections with *I* > 2σ(*I*)
graphite	*R*_int_ = 0.045
φ and ω scans	θ_max_ = 25.0°, θ_min_ = 2.4°
Absorption correction: multi-scan (*SADABS*; Bruker, 2007)	*h* = −13→14
*T*_min_ = 0.784, *T*_max_ = 0.875	*k* = −17→14
17415 measured reflections	*l* = −22→22

### Refinement

**Table:**

Refinement on *F*^2^	Primary atom site location: structure-invariant direct methods
Least-squares matrix: full	Secondary atom site location: difference Fourier map
*R*[*F*^2^ > 2σ(*F*^2^)] = 0.069	Hydrogen site location: inferred from neighbouring sites
*wR*(*F*^2^) = 0.215	H-atom parameters constrained
*S* = 1.03	*w* = 1/[σ^2^(*F*_o_^2^) + (0.1057*P*)^2^ + 0.2104*P*] where *P* = (*F*_o_^2^ + 2*F*_c_^2^)/3
11528 reflections	(Δ/σ)_max_ = 0.001
793 parameters	Δρ_max_ = 0.95 e Å^−^^3^
0 restraints	Δρ_min_ = −0.74 e Å^−^^3^

### Special details

**Table:**

Geometry. All e.s.d.'s (except the e.s.d. in the dihedral angle between two l.s. planes) are estimated using the full covariance matrix. The cell e.s.d.'s are taken into account individually in the estimation of e.s.d.'s in distances, angles and torsion angles; correlations between e.s.d.'s in cell parameters are only used when they are defined by crystal symmetry. An approximate (isotropic) treatment of cell e.s.d.'s is used for estimating e.s.d.'s involving l.s. planes.
Refinement. Refinement of *F*^2^ against ALL reflections. The weighted *R*-factor *wR* and goodness of fit *S* are based on *F*^2^, conventional *R*-factors *R* are based on *F*, with *F* set to zero for negative *F*^2^. The threshold expression of *F*^2^ > σ(*F*^2^) is used only for calculating *R*-factors(gt) *etc*. and is not relevant to the choice of reflections for refinement. *R*-factors based on *F*^2^ are statistically about twice as large as those based on *F*, and *R*- factors based on ALL data will be even larger.

### Fractional atomic coordinates and isotropic or equivalent isotropic displacement parameters (Å^2^)

**Table:**

	*x*	*y*	*z*	*U*_iso_*/*U*_eq_	
Ag1	0.49163 (4)	0.75389 (4)	0.25184 (3)	0.0419 (2)	
P1	0.39951 (14)	0.75380 (11)	0.38262 (9)	0.0354 (4)	
P2	0.56842 (15)	0.91843 (11)	0.21969 (9)	0.0369 (4)	
P3	0.34256 (15)	0.71082 (12)	0.16119 (9)	0.0396 (4)	
P4	0.65838 (15)	0.63223 (12)	0.23921 (9)	0.0390 (4)	
S1	0.9363 (2)	0.75400 (16)	0.65163 (15)	0.0724 (7)	
Cl1	0.0966 (5)	0.6655 (5)	0.9030 (3)	0.238 (3)	
Cl2	0.0693 (8)	0.8546 (5)	0.8966 (3)	0.305 (4)	
F3	1.1450 (6)	0.7157 (6)	0.6198 (4)	0.148 (3)	
F4	1.0369 (8)	0.6402 (6)	0.5650 (5)	0.174 (4)	
F5	1.0535 (7)	0.6115 (6)	0.6717 (5)	0.191 (4)	
O1	0.9448 (7)	0.8191 (6)	0.5993 (5)	0.150 (4)	
O2	0.9645 (7)	0.7799 (7)	0.7207 (5)	0.148 (3)	
O3	0.8415 (6)	0.7018 (5)	0.6538 (4)	0.106 (2)	
C1	0.2846 (6)	0.8358 (4)	0.3992 (3)	0.0385 (16)	
C2	0.2952 (6)	0.9215 (5)	0.3671 (4)	0.0500 (19)	
H2	0.3593	0.9347	0.3384	0.060*	
C3	0.2101 (7)	0.9864 (5)	0.3781 (4)	0.053 (2)	
H3	0.2178	1.0433	0.3569	0.064*	
C4	0.1163 (6)	0.9683 (5)	0.4192 (4)	0.056 (2)	
H4	0.0596	1.0123	0.4259	0.067*	
C5	0.1044 (6)	0.8844 (5)	0.4511 (4)	0.0520 (19)	
H5	0.0399	0.8719	0.4795	0.062*	
C6	0.1888 (6)	0.8183 (5)	0.4408 (4)	0.0447 (17)	
H6	0.1802	0.7617	0.4623	0.054*	
C7	0.4909 (6)	0.7786 (5)	0.4534 (3)	0.0402 (16)	
C8	0.4661 (7)	0.8421 (5)	0.5043 (4)	0.058 (2)	
H8	0.3988	0.8750	0.5048	0.069*	
C9	0.5410 (8)	0.8584 (6)	0.5561 (5)	0.075 (3)	
H9	0.5234	0.9022	0.5908	0.090*	
C10	0.6384 (7)	0.8112 (6)	0.5561 (4)	0.067 (2)	
H10	0.6876	0.8218	0.5910	0.081*	
C11	0.6651 (7)	0.7477 (6)	0.5049 (5)	0.067 (2)	
H11	0.7325	0.7151	0.5050	0.081*	
C12	0.5938 (6)	0.7318 (5)	0.4538 (4)	0.056 (2)	
H12	0.6137	0.6893	0.4185	0.068*	
C13	0.3348 (6)	0.6483 (5)	0.4134 (4)	0.0440 (17)	
C14	0.2682 (6)	0.6047 (5)	0.3669 (4)	0.056 (2)	
H14	0.2585	0.6293	0.3220	0.067*	
C15	0.2165 (7)	0.5251 (5)	0.3876 (4)	0.059 (2)	
H15	0.1721	0.4964	0.3565	0.071*	
C16	0.2298 (8)	0.4888 (6)	0.4521 (5)	0.072 (3)	
H16	0.1958	0.4345	0.4649	0.086*	
C17	0.2926 (8)	0.5306 (6)	0.4990 (5)	0.080 (3)	
H17	0.2983	0.5064	0.5445	0.095*	
C18	0.3491 (7)	0.6107 (5)	0.4787 (4)	0.060 (2)	
H18	0.3955	0.6375	0.5096	0.072*	
C19	0.4645 (6)	1.0100 (4)	0.2161 (3)	0.0404 (16)	
C20	0.4738 (7)	1.0954 (5)	0.2434 (4)	0.0533 (19)	
H20	0.5389	1.1092	0.2647	0.064*	
C21	0.3924 (8)	1.1604 (6)	0.2408 (5)	0.068 (2)	
H21	0.4036	1.2178	0.2586	0.081*	
C22	0.2939 (7)	1.1421 (6)	0.2121 (4)	0.060 (2)	
H22	0.2365	1.1857	0.2118	0.073*	
C23	0.2823 (7)	1.0585 (6)	0.1842 (4)	0.067 (2)	
H23	0.2170	1.0459	0.1627	0.081*	
C24	0.3636 (6)	0.9925 (5)	0.1867 (4)	0.058 (2)	
H24	0.3518	0.9353	0.1687	0.069*	
C25	0.6451 (6)	0.9263 (5)	0.1332 (4)	0.0456 (18)	
C26	0.6310 (7)	0.9963 (6)	0.0860 (4)	0.065 (2)	
H26	0.5769	1.0416	0.0966	0.078*	
C27	0.6982 (8)	0.9997 (7)	0.0216 (5)	0.089 (3)	
H27	0.6916	1.0490	−0.0090	0.107*	
C28	0.7725 (8)	0.9316 (7)	0.0042 (5)	0.079 (3)	
H28	0.8145	0.9332	−0.0394	0.095*	
C29	0.7866 (7)	0.8599 (6)	0.0501 (5)	0.074 (3)	
H29	0.8391	0.8139	0.0382	0.088*	
C30	0.7229 (6)	0.8569 (6)	0.1133 (4)	0.059 (2)	
H30	0.7314	0.8077	0.1438	0.070*	
C31	0.6674 (6)	0.9641 (5)	0.2791 (4)	0.0427 (17)	
C32	0.7568 (7)	1.0161 (5)	0.2537 (4)	0.061 (2)	
H32	0.7698	1.0270	0.2051	0.073*	
C33	0.8268 (7)	1.0518 (6)	0.3019 (5)	0.072 (3)	
H33	0.8866	1.0869	0.2853	0.086*	
C34	0.8080 (7)	1.0357 (6)	0.3733 (5)	0.069 (2)	
H34	0.8546	1.0603	0.4052	0.083*	
C35	0.7211 (7)	0.9834 (6)	0.3980 (4)	0.066 (2)	
H35	0.7092	0.9721	0.4467	0.079*	
C36	0.6502 (6)	0.9472 (5)	0.3510 (4)	0.0520 (19)	
H36	0.5912	0.9116	0.3681	0.062*	
C37	0.3954 (6)	0.7088 (5)	0.0677 (4)	0.0452 (18)	
C38	0.3656 (7)	0.6448 (6)	0.0200 (4)	0.065 (2)	
H38	0.3177	0.5989	0.0353	0.078*	
C39	0.4069 (8)	0.6487 (6)	−0.0509 (4)	0.079 (3)	
H39	0.3878	0.6048	−0.0829	0.095*	
C40	0.4752 (8)	0.7165 (6)	−0.0734 (5)	0.074 (3)	
H40	0.5012	0.7197	−0.1211	0.089*	
C41	0.5065 (7)	0.7807 (6)	−0.0263 (5)	0.068 (2)	
H41	0.5548	0.8262	−0.0419	0.082*	
C42	0.4665 (6)	0.7769 (5)	0.0429 (4)	0.055 (2)	
H42	0.4870	0.8207	0.0744	0.066*	
C43	0.2189 (6)	0.7849 (5)	0.1592 (4)	0.0443 (17)	
C44	0.1698 (7)	0.8111 (6)	0.0981 (4)	0.060 (2)	
H44	0.2011	0.7905	0.0542	0.072*	
C45	0.0769 (7)	0.8662 (6)	0.1000 (5)	0.075 (3)	
H45	0.0463	0.8832	0.0577	0.090*	
C46	0.0291 (7)	0.8964 (6)	0.1628 (5)	0.074 (3)	
H46	−0.0353	0.9331	0.1640	0.089*	
C47	0.0753 (7)	0.8729 (6)	0.2239 (5)	0.074 (3)	
H47	0.0428	0.8944	0.2671	0.089*	
C48	0.1697 (7)	0.8178 (6)	0.2232 (4)	0.062 (2)	
H48	0.2007	0.8026	0.2658	0.074*	
C49	0.2821 (6)	0.5995 (5)	0.1743 (4)	0.0461 (18)	
C50	0.1691 (7)	0.5851 (5)	0.1785 (4)	0.059 (2)	
H50	0.1196	0.6340	0.1737	0.071*	
C51	0.1274 (8)	0.4988 (6)	0.1899 (5)	0.071 (2)	
H51	0.0506	0.4902	0.1922	0.085*	
C52	0.1992 (8)	0.4266 (6)	0.1977 (4)	0.069 (2)	
H52	0.1713	0.3689	0.2056	0.083*	
C53	0.3107 (8)	0.4389 (6)	0.1940 (5)	0.071 (2)	
H53	0.3596	0.3897	0.1991	0.086*	
C54	0.3522 (7)	0.5253 (5)	0.1826 (4)	0.062 (2)	
H54	0.4291	0.5331	0.1805	0.074*	
C55	0.7317 (6)	0.6186 (5)	0.1513 (4)	0.0457 (18)	
C56	0.6690 (7)	0.6049 (5)	0.0936 (4)	0.055 (2)	
H56	0.5915	0.6034	0.1006	0.066*	
C57	0.7193 (8)	0.5935 (6)	0.0260 (4)	0.068 (2)	
H57	0.6756	0.5861	−0.0124	0.082*	
C58	0.8317 (8)	0.5930 (6)	0.0152 (4)	0.074 (3)	
H58	0.8654	0.5828	−0.0303	0.089*	
C59	0.8966 (8)	0.6075 (7)	0.0713 (5)	0.084 (3)	
H59	0.9741	0.6092	0.0638	0.100*	
C60	0.8451 (7)	0.6195 (6)	0.1387 (4)	0.067 (2)	
H60	0.8890	0.6286	0.1767	0.081*	
C61	0.7708 (6)	0.6546 (5)	0.2966 (4)	0.0422 (17)	
C62	0.7971 (7)	0.7423 (5)	0.3089 (4)	0.061 (2)	
H62	0.7569	0.7891	0.2884	0.074*	
C63	0.8823 (7)	0.7627 (6)	0.3515 (5)	0.074 (3)	
H63	0.9009	0.8228	0.3580	0.089*	
C64	0.9390 (7)	0.6947 (7)	0.3839 (5)	0.075 (3)	
H64	0.9944	0.7082	0.4141	0.090*	
C65	0.9144 (7)	0.6070 (6)	0.3719 (5)	0.072 (3)	
H65	0.9544	0.5605	0.3931	0.087*	
C66	0.8305 (7)	0.5864 (6)	0.3287 (4)	0.060 (2)	
H66	0.8141	0.5261	0.3211	0.072*	
C67	0.6223 (6)	0.5157 (5)	0.2643 (4)	0.0450 (17)	
C68	0.5422 (7)	0.5028 (5)	0.3194 (4)	0.059 (2)	
H68	0.5033	0.5527	0.3397	0.071*	
C69	0.5190 (8)	0.4182 (6)	0.3447 (5)	0.071 (2)	
H69	0.4665	0.4114	0.3832	0.085*	
C70	0.5706 (8)	0.3449 (6)	0.3152 (5)	0.075 (3)	
H70	0.5517	0.2874	0.3318	0.090*	
C71	0.6526 (8)	0.3546 (6)	0.2594 (5)	0.077 (3)	
H71	0.6909	0.3040	0.2399	0.093*	
C72	0.6765 (7)	0.4413 (5)	0.2332 (4)	0.060 (2)	
H72	0.7290	0.4485	0.1948	0.072*	
C73	1.0507 (11)	0.6801 (9)	0.6259 (8)	0.107 (4)	
C74	0.021 (2)	0.7551 (12)	0.8686 (10)	0.239 (11)	
H74A	0.0273	0.7529	0.8168	0.287*	
H74B	−0.0579	0.7503	0.8844	0.287*	

### Atomic displacement parameters (Å^2^)

**Table:**

	*U*^11^	*U*^22^	*U*^33^	*U*^12^	*U*^13^	*U*^23^
Ag1	0.0406 (4)	0.0455 (3)	0.0395 (3)	−0.0003 (2)	−0.0030 (2)	−0.0005 (2)
P1	0.0369 (10)	0.0380 (10)	0.0305 (9)	0.0007 (8)	0.0014 (7)	0.0003 (7)
P2	0.0383 (10)	0.0345 (10)	0.0381 (10)	−0.0051 (8)	−0.0022 (8)	0.0016 (7)
P3	0.0363 (10)	0.0468 (11)	0.0367 (10)	−0.0035 (8)	−0.0088 (8)	−0.0026 (8)
P4	0.0325 (10)	0.0426 (11)	0.0417 (10)	0.0078 (8)	−0.0032 (8)	−0.0015 (8)
S1	0.0617 (15)	0.0602 (15)	0.0944 (19)	−0.0011 (12)	0.0015 (13)	0.0012 (13)
Cl1	0.212 (6)	0.274 (7)	0.221 (6)	0.033 (5)	0.027 (5)	0.006 (5)
Cl2	0.497 (13)	0.254 (7)	0.164 (5)	−0.104 (8)	0.010 (6)	0.038 (5)
F3	0.068 (4)	0.170 (7)	0.201 (8)	0.011 (5)	0.016 (5)	0.002 (6)
F4	0.179 (8)	0.167 (8)	0.169 (8)	0.012 (6)	0.031 (6)	−0.083 (7)
F5	0.175 (8)	0.138 (7)	0.255 (11)	0.064 (6)	−0.011 (7)	0.077 (7)
O1	0.128 (7)	0.118 (7)	0.194 (9)	0.027 (5)	0.028 (6)	0.092 (6)
O2	0.113 (7)	0.198 (9)	0.134 (7)	−0.021 (6)	−0.003 (6)	−0.074 (6)
O3	0.071 (5)	0.112 (6)	0.140 (7)	−0.026 (4)	−0.035 (4)	0.020 (5)
C1	0.043 (4)	0.038 (4)	0.035 (4)	0.000 (3)	−0.005 (3)	0.000 (3)
C2	0.050 (5)	0.051 (5)	0.047 (4)	0.002 (4)	0.005 (4)	0.006 (4)
C3	0.062 (5)	0.039 (4)	0.057 (5)	0.010 (4)	0.002 (4)	0.002 (4)
C4	0.042 (5)	0.061 (5)	0.065 (5)	0.017 (4)	−0.005 (4)	−0.018 (4)
C5	0.040 (4)	0.053 (5)	0.061 (5)	−0.004 (4)	0.009 (4)	−0.011 (4)
C6	0.043 (4)	0.045 (4)	0.045 (4)	0.001 (3)	0.006 (3)	0.002 (3)
C7	0.037 (4)	0.043 (4)	0.040 (4)	0.000 (3)	−0.003 (3)	0.002 (3)
C8	0.057 (5)	0.068 (5)	0.050 (5)	0.013 (4)	−0.013 (4)	−0.015 (4)
C9	0.079 (7)	0.085 (7)	0.065 (6)	0.008 (6)	−0.024 (5)	−0.029 (5)
C10	0.057 (6)	0.081 (6)	0.067 (6)	−0.002 (5)	−0.030 (5)	−0.010 (5)
C11	0.055 (5)	0.074 (6)	0.074 (6)	0.012 (5)	−0.021 (5)	−0.001 (5)
C12	0.053 (5)	0.063 (5)	0.054 (5)	0.009 (4)	−0.008 (4)	−0.009 (4)
C13	0.052 (5)	0.041 (4)	0.039 (4)	−0.001 (3)	−0.003 (3)	−0.002 (3)
C14	0.062 (5)	0.055 (5)	0.050 (5)	−0.009 (4)	−0.008 (4)	0.002 (4)
C15	0.060 (5)	0.053 (5)	0.066 (6)	−0.012 (4)	−0.012 (4)	−0.008 (4)
C16	0.081 (7)	0.050 (5)	0.085 (7)	−0.023 (5)	−0.004 (5)	0.011 (5)
C17	0.099 (8)	0.067 (6)	0.073 (6)	−0.021 (6)	−0.009 (6)	0.023 (5)
C18	0.078 (6)	0.055 (5)	0.048 (5)	−0.016 (4)	−0.009 (4)	0.013 (4)
C19	0.041 (4)	0.042 (4)	0.038 (4)	−0.003 (3)	0.002 (3)	0.001 (3)
C20	0.052 (5)	0.050 (5)	0.059 (5)	0.001 (4)	−0.009 (4)	−0.005 (4)
C21	0.075 (6)	0.053 (5)	0.074 (6)	0.017 (5)	−0.008 (5)	0.001 (4)
C22	0.056 (6)	0.065 (6)	0.059 (5)	0.017 (4)	0.000 (4)	0.010 (4)
C23	0.054 (5)	0.077 (6)	0.071 (6)	0.006 (5)	−0.016 (4)	0.003 (5)
C24	0.051 (5)	0.056 (5)	0.067 (5)	0.001 (4)	−0.009 (4)	0.001 (4)
C25	0.044 (4)	0.048 (4)	0.045 (4)	−0.007 (4)	0.000 (3)	0.003 (3)
C26	0.067 (6)	0.070 (6)	0.056 (5)	0.016 (4)	0.016 (4)	0.017 (4)
C27	0.091 (8)	0.105 (8)	0.067 (6)	0.015 (6)	0.025 (6)	0.034 (6)
C28	0.083 (7)	0.099 (8)	0.053 (6)	0.008 (6)	0.024 (5)	0.002 (5)
C29	0.057 (6)	0.087 (7)	0.074 (6)	0.014 (5)	0.014 (5)	−0.003 (5)
C30	0.053 (5)	0.070 (6)	0.051 (5)	0.002 (4)	0.008 (4)	0.010 (4)
C31	0.045 (4)	0.042 (4)	0.042 (4)	−0.004 (3)	−0.004 (3)	−0.002 (3)
C32	0.056 (5)	0.069 (6)	0.057 (5)	−0.015 (4)	−0.001 (4)	−0.004 (4)
C33	0.055 (5)	0.085 (7)	0.077 (7)	−0.025 (5)	−0.006 (5)	−0.013 (5)
C34	0.063 (6)	0.079 (6)	0.068 (6)	−0.003 (5)	−0.027 (5)	−0.015 (5)
C35	0.073 (6)	0.073 (6)	0.054 (5)	−0.001 (5)	−0.014 (5)	−0.004 (4)
C36	0.055 (5)	0.054 (5)	0.049 (5)	−0.007 (4)	−0.011 (4)	0.004 (4)
C37	0.042 (4)	0.052 (5)	0.042 (4)	0.000 (4)	−0.007 (3)	−0.002 (3)
C38	0.077 (6)	0.068 (6)	0.051 (5)	−0.016 (5)	−0.002 (4)	−0.005 (4)
C39	0.106 (8)	0.082 (7)	0.050 (6)	−0.015 (6)	0.001 (5)	−0.018 (5)
C40	0.086 (7)	0.081 (7)	0.052 (5)	0.000 (6)	0.014 (5)	0.002 (5)
C41	0.069 (6)	0.073 (6)	0.061 (6)	−0.009 (5)	0.011 (5)	0.009 (5)
C42	0.053 (5)	0.062 (5)	0.049 (5)	−0.004 (4)	−0.001 (4)	−0.004 (4)
C43	0.041 (4)	0.052 (5)	0.041 (4)	−0.004 (3)	−0.010 (3)	−0.002 (3)
C44	0.052 (5)	0.075 (6)	0.055 (5)	0.007 (4)	−0.010 (4)	0.000 (4)
C45	0.051 (6)	0.101 (7)	0.072 (6)	0.018 (5)	−0.015 (5)	0.010 (5)
C46	0.053 (6)	0.086 (7)	0.083 (7)	0.013 (5)	−0.004 (5)	−0.005 (5)
C47	0.060 (6)	0.089 (7)	0.072 (6)	0.010 (5)	0.007 (5)	−0.016 (5)
C48	0.058 (5)	0.072 (6)	0.055 (5)	0.008 (5)	−0.005 (4)	−0.007 (4)
C49	0.052 (5)	0.047 (4)	0.040 (4)	−0.002 (4)	−0.007 (3)	0.000 (3)
C50	0.057 (5)	0.052 (5)	0.071 (6)	−0.006 (4)	−0.013 (4)	0.006 (4)
C51	0.057 (6)	0.070 (6)	0.086 (7)	−0.021 (5)	−0.005 (5)	0.012 (5)
C52	0.086 (7)	0.051 (5)	0.072 (6)	−0.017 (5)	−0.007 (5)	0.012 (4)
C53	0.076 (7)	0.060 (6)	0.078 (6)	0.011 (5)	−0.007 (5)	0.007 (5)
C54	0.060 (5)	0.059 (6)	0.067 (6)	−0.002 (4)	−0.005 (4)	0.004 (4)
C55	0.043 (5)	0.049 (5)	0.044 (4)	0.010 (4)	−0.003 (3)	0.001 (3)
C56	0.050 (5)	0.069 (5)	0.046 (5)	0.006 (4)	−0.001 (4)	0.000 (4)
C57	0.073 (7)	0.082 (6)	0.050 (5)	0.009 (5)	−0.009 (5)	−0.005 (4)
C58	0.075 (7)	0.100 (7)	0.044 (5)	0.014 (6)	0.012 (5)	−0.002 (5)
C59	0.060 (6)	0.121 (9)	0.066 (6)	0.011 (6)	0.015 (5)	−0.001 (6)
C60	0.056 (6)	0.091 (7)	0.054 (5)	0.008 (5)	−0.007 (4)	−0.001 (5)
C61	0.037 (4)	0.046 (4)	0.044 (4)	0.003 (3)	−0.005 (3)	−0.002 (3)
C62	0.061 (5)	0.055 (5)	0.070 (6)	0.002 (4)	−0.023 (4)	0.003 (4)
C63	0.067 (6)	0.066 (6)	0.094 (7)	−0.011 (5)	−0.023 (5)	−0.006 (5)
C64	0.060 (6)	0.084 (7)	0.085 (7)	−0.012 (5)	−0.031 (5)	−0.007 (5)
C65	0.063 (6)	0.068 (6)	0.089 (7)	0.004 (5)	−0.030 (5)	0.011 (5)
C66	0.054 (5)	0.055 (5)	0.073 (6)	−0.001 (4)	−0.018 (4)	0.002 (4)
C67	0.041 (4)	0.043 (4)	0.051 (5)	0.005 (3)	−0.008 (4)	−0.002 (3)
C68	0.059 (5)	0.050 (5)	0.067 (5)	0.003 (4)	0.000 (4)	0.003 (4)
C69	0.071 (6)	0.072 (7)	0.069 (6)	−0.010 (5)	−0.004 (5)	0.012 (5)
C70	0.088 (7)	0.060 (6)	0.080 (7)	−0.015 (5)	−0.017 (6)	0.008 (5)
C71	0.093 (8)	0.052 (6)	0.087 (7)	−0.002 (5)	−0.011 (6)	−0.007 (5)
C72	0.067 (6)	0.053 (5)	0.058 (5)	−0.002 (4)	0.005 (4)	−0.005 (4)
C73	0.094 (10)	0.091 (9)	0.135 (11)	0.014 (7)	0.000 (8)	0.033 (8)
C74	0.37 (3)	0.158 (17)	0.20 (2)	−0.01 (2)	−0.13 (2)	−0.020 (14)

### Geometric parameters (Å, °)

**Table:**

Ag1—P1	2.6202 (17)	C31—C36	1.374 (9)
Ag1—P3	2.6480 (17)	C31—C32	1.389 (10)
Ag1—P4	2.6494 (18)	C32—C33	1.393 (10)
Ag1—P2	2.6682 (17)	C32—H32	0.9300
P1—C1	1.820 (7)	C33—C34	1.364 (11)
P1—C7	1.828 (7)	C33—H33	0.9300
P1—C13	1.829 (7)	C34—C35	1.365 (11)
P2—C25	1.813 (7)	C34—H34	0.9300
P2—C19	1.818 (7)	C35—C36	1.388 (10)
P2—C31	1.830 (7)	C35—H35	0.9300
P3—C49	1.820 (7)	C36—H36	0.9300
P3—C43	1.820 (7)	C37—C38	1.377 (10)
P3—C37	1.824 (7)	C37—C42	1.391 (10)
P4—C61	1.823 (7)	C38—C39	1.388 (11)
P4—C55	1.823 (7)	C38—H38	0.9300
P4—C67	1.833 (7)	C39—C40	1.355 (12)
S1—O1	1.370 (7)	C39—H39	0.9300
S1—O3	1.388 (6)	C40—C41	1.377 (11)
S1—O2	1.421 (8)	C40—H40	0.9300
S1—C73	1.771 (13)	C41—C42	1.354 (10)
Cl1—C74	1.734 (18)	C41—H41	0.9300
Cl2—C74	1.694 (17)	C42—H42	0.9300
F3—C73	1.256 (13)	C43—C44	1.375 (9)
F4—C73	1.314 (14)	C43—C48	1.387 (10)
F5—C73	1.325 (12)	C44—C45	1.360 (11)
C1—C6	1.370 (9)	C44—H44	0.9300
C1—C2	1.402 (9)	C45—C46	1.347 (12)
C2—C3	1.387 (10)	C45—H45	0.9300
C2—H2	0.9300	C46—C47	1.349 (11)
C3—C4	1.349 (10)	C46—H46	0.9300
C3—H3	0.9300	C47—C48	1.375 (11)
C4—C5	1.378 (10)	C47—H47	0.9300
C4—H4	0.9300	C48—H48	0.9300
C5—C6	1.390 (10)	C49—C50	1.373 (10)
C5—H5	0.9300	C49—C54	1.378 (10)
C6—H6	0.9300	C50—C51	1.390 (10)
C7—C8	1.355 (9)	C50—H50	0.9300
C7—C12	1.397 (10)	C51—C52	1.365 (12)
C8—C9	1.397 (10)	C51—H51	0.9300
C8—H8	0.9300	C52—C53	1.349 (12)
C9—C10	1.343 (11)	C52—H52	0.9300
C9—H9	0.9300	C53—C54	1.390 (11)
C10—C11	1.362 (11)	C53—H53	0.9300
C10—H10	0.9300	C54—H54	0.9300
C11—C12	1.356 (10)	C55—C60	1.364 (10)
C11—H11	0.9300	C55—C56	1.382 (9)
C12—H12	0.9300	C56—C57	1.375 (10)
C13—C18	1.363 (9)	C56—H56	0.9300
C13—C14	1.396 (9)	C57—C58	1.348 (11)
C14—C15	1.383 (10)	C57—H57	0.9300
C14—H14	0.9300	C58—C59	1.376 (12)
C15—C16	1.339 (11)	C58—H58	0.9300
C15—H15	0.9300	C59—C60	1.379 (11)
C16—C17	1.361 (11)	C59—H59	0.9300
C16—H16	0.9300	C60—H60	0.9300
C17—C18	1.414 (10)	C61—C62	1.364 (10)
C17—H17	0.9300	C61—C66	1.380 (9)
C18—H18	0.9300	C62—C63	1.382 (10)
C19—C20	1.374 (9)	C62—H62	0.9300
C19—C24	1.396 (10)	C63—C64	1.362 (11)
C20—C21	1.352 (10)	C63—H63	0.9300
C20—H20	0.9300	C64—C65	1.358 (11)
C21—C22	1.366 (11)	C64—H64	0.9300
C21—H21	0.9300	C65—C66	1.378 (10)
C22—C23	1.359 (11)	C65—H65	0.9300
C22—H22	0.9300	C66—H66	0.9300
C23—C24	1.362 (11)	C67—C72	1.373 (10)
C23—H23	0.9300	C67—C68	1.377 (10)
C24—H24	0.9300	C68—C69	1.360 (10)
C25—C26	1.372 (10)	C68—H68	0.9300
C25—C30	1.401 (10)	C69—C70	1.335 (12)
C26—C27	1.405 (11)	C69—H69	0.9300
C26—H26	0.9300	C70—C71	1.393 (13)
C27—C28	1.351 (12)	C70—H70	0.9300
C27—H27	0.9300	C71—C72	1.396 (11)
C28—C29	1.374 (12)	C71—H71	0.9300
C28—H28	0.9300	C72—H72	0.9300
C29—C30	1.367 (11)	C74—H74A	0.9700
C29—H29	0.9300	C74—H74B	0.9700
C30—H30	0.9300		
			
P1—Ag1—P3	110.10 (6)	C34—C33—C32	120.4 (8)
P1—Ag1—P4	110.15 (6)	C34—C33—H33	119.8
P3—Ag1—P4	108.02 (6)	C32—C33—H33	119.8
P1—Ag1—P2	109.41 (6)	C33—C34—C35	120.2 (8)
P3—Ag1—P2	109.41 (6)	C33—C34—H34	119.9
P4—Ag1—P2	109.74 (6)	C35—C34—H34	119.9
C1—P1—C7	102.6 (3)	C34—C35—C36	120.4 (8)
C1—P1—C13	101.7 (3)	C34—C35—H35	119.8
C7—P1—C13	102.9 (3)	C36—C35—H35	119.8
C1—P1—Ag1	114.8 (2)	C31—C36—C35	119.9 (7)
C7—P1—Ag1	116.6 (2)	C31—C36—H36	120.0
C13—P1—Ag1	116.2 (2)	C35—C36—H36	120.0
C25—P2—C19	102.7 (3)	C38—C37—C42	118.4 (7)
C25—P2—C31	102.1 (3)	C38—C37—P3	123.2 (6)
C19—P2—C31	102.5 (3)	C42—C37—P3	118.4 (5)
C25—P2—Ag1	114.4 (2)	C37—C38—C39	120.2 (8)
C19—P2—Ag1	116.6 (2)	C37—C38—H38	119.9
C31—P2—Ag1	116.5 (2)	C39—C38—H38	119.9
C49—P3—C43	102.3 (3)	C40—C39—C38	119.8 (8)
C49—P3—C37	103.0 (3)	C40—C39—H39	120.1
C43—P3—C37	102.4 (3)	C38—C39—H39	120.1
C49—P3—Ag1	115.6 (2)	C39—C40—C41	120.8 (8)
C43—P3—Ag1	116.7 (2)	C39—C40—H40	119.6
C37—P3—Ag1	114.8 (2)	C41—C40—H40	119.6
C61—P4—C55	103.2 (3)	C42—C41—C40	119.4 (8)
C61—P4—C67	102.2 (3)	C42—C41—H41	120.3
C55—P4—C67	102.9 (3)	C40—C41—H41	120.3
C61—P4—Ag1	113.4 (2)	C41—C42—C37	121.4 (7)
C55—P4—Ag1	117.4 (2)	C41—C42—H42	119.3
C67—P4—Ag1	115.7 (2)	C37—C42—H42	119.3
O1—S1—O3	116.2 (5)	C44—C43—C48	116.9 (7)
O1—S1—O2	116.6 (6)	C44—C43—P3	124.6 (6)
O3—S1—O2	112.6 (5)	C48—C43—P3	118.5 (5)
O1—S1—C73	101.8 (6)	C45—C44—C43	121.9 (8)
O3—S1—C73	106.3 (6)	C45—C44—H44	119.0
O2—S1—C73	100.7 (6)	C43—C44—H44	119.0
C6—C1—C2	118.5 (6)	C46—C45—C44	120.4 (8)
C6—C1—P1	123.8 (5)	C46—C45—H45	119.8
C2—C1—P1	117.6 (5)	C44—C45—H45	119.8
C3—C2—C1	119.9 (7)	C45—C46—C47	119.5 (9)
C3—C2—H2	120.0	C45—C46—H46	120.3
C1—C2—H2	120.0	C47—C46—H46	120.3
C4—C3—C2	120.9 (7)	C46—C47—C48	121.2 (9)
C4—C3—H3	119.6	C46—C47—H47	119.4
C2—C3—H3	119.6	C48—C47—H47	119.4
C3—C4—C5	119.9 (7)	C47—C48—C43	120.1 (8)
C3—C4—H4	120.0	C47—C48—H48	119.9
C5—C4—H4	120.0	C43—C48—H48	119.9
C4—C5—C6	120.1 (7)	C50—C49—C54	117.4 (7)
C4—C5—H5	120.0	C50—C49—P3	123.6 (6)
C6—C5—H5	120.0	C54—C49—P3	119.0 (6)
C1—C6—C5	120.6 (7)	C49—C50—C51	121.2 (8)
C1—C6—H6	119.7	C49—C50—H50	119.4
C5—C6—H6	119.7	C51—C50—H50	119.4
C8—C7—C12	118.0 (7)	C52—C51—C50	119.9 (8)
C8—C7—P1	123.6 (6)	C52—C51—H51	120.0
C12—C7—P1	118.3 (5)	C50—C51—H51	120.0
C7—C8—C9	120.4 (8)	C53—C52—C51	120.1 (8)
C7—C8—H8	119.8	C53—C52—H52	119.9
C9—C8—H8	119.8	C51—C52—H52	119.9
C10—C9—C8	120.4 (8)	C52—C53—C54	119.9 (8)
C10—C9—H9	119.8	C52—C53—H53	120.1
C8—C9—H9	119.8	C54—C53—H53	120.1
C9—C10—C11	120.0 (7)	C49—C54—C53	121.5 (8)
C9—C10—H10	120.0	C49—C54—H54	119.3
C11—C10—H10	120.0	C53—C54—H54	119.3
C12—C11—C10	120.3 (8)	C60—C55—C56	117.4 (7)
C12—C11—H11	119.8	C60—C55—P4	124.3 (6)
C10—C11—H11	119.8	C56—C55—P4	118.3 (6)
C11—C12—C7	120.9 (7)	C57—C56—C55	121.2 (8)
C11—C12—H12	119.6	C57—C56—H56	119.4
C7—C12—H12	119.6	C55—C56—H56	119.4
C18—C13—C14	119.2 (7)	C58—C57—C56	120.2 (8)
C18—C13—P1	123.2 (6)	C58—C57—H57	119.9
C14—C13—P1	117.6 (5)	C56—C57—H57	119.9
C15—C14—C13	120.0 (7)	C57—C58—C59	120.2 (8)
C15—C14—H14	120.0	C57—C58—H58	119.9
C13—C14—H14	120.0	C59—C58—H58	119.9
C16—C15—C14	120.7 (7)	C58—C59—C60	119.0 (9)
C16—C15—H15	119.7	C58—C59—H59	120.5
C14—C15—H15	119.7	C60—C59—H59	120.5
C15—C16—C17	120.6 (8)	C55—C60—C59	122.0 (8)
C15—C16—H16	119.7	C55—C60—H60	119.0
C17—C16—H16	119.7	C59—C60—H60	119.0
C16—C17—C18	120.1 (8)	C62—C61—C66	118.2 (7)
C16—C17—H17	120.0	C62—C61—P4	119.1 (6)
C18—C17—H17	120.0	C66—C61—P4	122.8 (6)
C13—C18—C17	119.4 (7)	C61—C62—C63	121.2 (7)
C13—C18—H18	120.3	C61—C62—H62	119.4
C17—C18—H18	120.3	C63—C62—H62	119.4
C20—C19—C24	115.9 (7)	C64—C63—C62	119.8 (8)
C20—C19—P2	125.5 (6)	C64—C63—H63	120.1
C24—C19—P2	118.5 (5)	C62—C63—H63	120.1
C21—C20—C19	122.9 (8)	C65—C64—C63	119.7 (8)
C21—C20—H20	118.5	C65—C64—H64	120.1
C19—C20—H20	118.5	C63—C64—H64	120.1
C20—C21—C22	120.5 (8)	C64—C65—C66	120.5 (8)
C20—C21—H21	119.8	C64—C65—H65	119.8
C22—C21—H21	119.8	C66—C65—H65	119.8
C23—C22—C21	118.1 (8)	C65—C66—C61	120.5 (7)
C23—C22—H22	121.0	C65—C66—H66	119.7
C21—C22—H22	121.0	C61—C66—H66	119.7
C22—C23—C24	121.9 (8)	C72—C67—C68	118.8 (7)
C22—C23—H23	119.1	C72—C67—P4	122.7 (6)
C24—C23—H23	119.1	C68—C67—P4	118.4 (6)
C23—C24—C19	120.6 (8)	C69—C68—C67	121.1 (8)
C23—C24—H24	119.7	C69—C68—H68	119.4
C19—C24—H24	119.7	C67—C68—H68	119.4
C26—C25—C30	118.1 (7)	C70—C69—C68	121.0 (9)
C26—C25—P2	123.7 (6)	C70—C69—H69	119.5
C30—C25—P2	118.2 (5)	C68—C69—H69	119.5
C25—C26—C27	120.0 (8)	C69—C70—C71	120.0 (9)
C25—C26—H26	120.0	C69—C70—H70	120.0
C27—C26—H26	120.0	C71—C70—H70	120.0
C28—C27—C26	120.2 (8)	C70—C71—C72	119.2 (9)
C28—C27—H27	119.9	C70—C71—H71	120.4
C26—C27—H27	119.9	C72—C71—H71	120.4
C27—C28—C29	120.8 (8)	C67—C72—C71	119.8 (8)
C27—C28—H28	119.6	C67—C72—H72	120.1
C29—C28—H28	119.6	C71—C72—H72	120.1
C30—C29—C28	119.4 (8)	F3—C73—F4	106.9 (12)
C30—C29—H29	120.3	F3—C73—F5	109.3 (12)
C28—C29—H29	120.3	F4—C73—F5	103.3 (12)
C29—C30—C25	121.4 (7)	F3—C73—S1	115.8 (10)
C29—C30—H30	119.3	F4—C73—S1	111.5 (10)
C25—C30—H30	119.3	F5—C73—S1	109.3 (10)
C36—C31—C32	119.8 (7)	Cl2—C74—Cl1	109.6 (11)
C36—C31—P2	118.2 (5)	Cl2—C74—H74A	109.7
C32—C31—P2	122.0 (5)	Cl1—C74—H74A	109.7
C31—C32—C33	119.3 (7)	Cl2—C74—H74B	109.7
C31—C32—H32	120.3	Cl1—C74—H74B	109.7
C33—C32—H32	120.3	H74A—C74—H74B	108.2
			
P3—Ag1—P1—C1	−61.9 (2)	C19—P2—C31—C36	90.6 (6)
P4—Ag1—P1—C1	179.1 (2)	Ag1—P2—C31—C36	−37.9 (7)
P2—Ag1—P1—C1	58.4 (2)	C25—P2—C31—C32	18.0 (7)
P3—Ag1—P1—C7	178.1 (2)	C19—P2—C31—C32	−88.1 (7)
P4—Ag1—P1—C7	59.1 (3)	Ag1—P2—C31—C32	143.4 (6)
P2—Ag1—P1—C7	−61.6 (3)	C36—C31—C32—C33	−1.1 (12)
P3—Ag1—P1—C13	56.6 (3)	P2—C31—C32—C33	177.6 (6)
P4—Ag1—P1—C13	−62.4 (3)	C31—C32—C33—C34	0.2 (13)
P2—Ag1—P1—C13	176.9 (3)	C32—C33—C34—C35	0.7 (14)
P1—Ag1—P2—C25	175.1 (3)	C33—C34—C35—C36	−0.8 (14)
P3—Ag1—P2—C25	−64.3 (3)	C32—C31—C36—C35	1.0 (11)
P4—Ag1—P2—C25	54.1 (3)	P2—C31—C36—C35	−177.6 (6)
P1—Ag1—P2—C19	−65.2 (2)	C34—C35—C36—C31	−0.1 (12)
P3—Ag1—P2—C19	55.5 (2)	C49—P3—C37—C38	14.4 (7)
P4—Ag1—P2—C19	173.8 (2)	C43—P3—C37—C38	−91.6 (7)
P1—Ag1—P2—C31	56.1 (3)	Ag1—P3—C37—C38	140.9 (6)
P3—Ag1—P2—C31	176.8 (3)	C49—P3—C37—C42	−167.7 (6)
P4—Ag1—P2—C31	−64.8 (3)	C43—P3—C37—C42	86.4 (6)
P1—Ag1—P3—C49	−60.5 (3)	Ag1—P3—C37—C42	−41.1 (6)
P4—Ag1—P3—C49	59.8 (3)	C42—C37—C38—C39	0.4 (12)
P2—Ag1—P3—C49	179.2 (3)	P3—C37—C38—C39	178.4 (7)
P1—Ag1—P3—C43	59.9 (3)	C37—C38—C39—C40	−1.1 (14)
P4—Ag1—P3—C43	−179.8 (3)	C38—C39—C40—C41	1.6 (15)
P2—Ag1—P3—C43	−60.4 (3)	C39—C40—C41—C42	−1.5 (14)
P1—Ag1—P3—C37	179.7 (3)	C40—C41—C42—C37	0.8 (13)
P4—Ag1—P3—C37	−60.0 (3)	C38—C37—C42—C41	−0.3 (12)
P2—Ag1—P3—C37	59.4 (3)	P3—C37—C42—C41	−178.4 (6)
P1—Ag1—P4—C61	−61.5 (3)	C49—P3—C43—C44	−93.0 (7)
P3—Ag1—P4—C61	178.2 (2)	C37—P3—C43—C44	13.4 (7)
P2—Ag1—P4—C61	59.0 (2)	Ag1—P3—C43—C44	139.7 (6)
P1—Ag1—P4—C55	178.2 (3)	C49—P3—C43—C48	87.0 (6)
P3—Ag1—P4—C55	57.9 (3)	C37—P3—C43—C48	−166.5 (6)
P2—Ag1—P4—C55	−61.3 (3)	Ag1—P3—C43—C48	−40.2 (7)
P1—Ag1—P4—C67	56.1 (3)	C48—C43—C44—C45	−0.4 (12)
P3—Ag1—P4—C67	−64.1 (3)	P3—C43—C44—C45	179.6 (7)
P2—Ag1—P4—C67	176.7 (2)	C43—C44—C45—C46	−0.7 (14)
C7—P1—C1—C6	−92.2 (6)	C44—C45—C46—C47	1.3 (15)
C13—P1—C1—C6	14.1 (6)	C45—C46—C47—C48	−0.8 (15)
Ag1—P1—C1—C6	140.4 (5)	C46—C47—C48—C43	−0.3 (14)
C7—P1—C1—C2	88.2 (6)	C44—C43—C48—C47	0.9 (12)
C13—P1—C1—C2	−165.6 (5)	P3—C43—C48—C47	−179.1 (7)
Ag1—P1—C1—C2	−39.3 (6)	C43—P3—C49—C50	1.7 (7)
C6—C1—C2—C3	0.5 (10)	C37—P3—C49—C50	−104.3 (7)
P1—C1—C2—C3	−179.9 (6)	Ag1—P3—C49—C50	129.6 (6)
C1—C2—C3—C4	−0.4 (11)	C43—P3—C49—C54	−176.4 (6)
C2—C3—C4—C5	0.3 (12)	C37—P3—C49—C54	77.6 (6)
C3—C4—C5—C6	−0.3 (11)	Ag1—P3—C49—C54	−48.4 (6)
C2—C1—C6—C5	−0.4 (10)	C54—C49—C50—C51	−0.7 (11)
P1—C1—C6—C5	180.0 (5)	P3—C49—C50—C51	−178.8 (6)
C4—C5—C6—C1	0.3 (11)	C49—C50—C51—C52	0.6 (13)
C1—P1—C7—C8	2.5 (7)	C50—C51—C52—C53	−0.4 (13)
C13—P1—C7—C8	−102.9 (7)	C51—C52—C53—C54	0.3 (14)
Ag1—P1—C7—C8	128.8 (6)	C50—C49—C54—C53	0.7 (11)
C1—P1—C7—C12	−175.3 (6)	P3—C49—C54—C53	178.8 (6)
C13—P1—C7—C12	79.4 (6)	C52—C53—C54—C49	−0.5 (13)
Ag1—P1—C7—C12	−48.9 (6)	C61—P4—C55—C60	4.2 (8)
C12—C7—C8—C9	−1.2 (12)	C67—P4—C55—C60	−101.9 (7)
P1—C7—C8—C9	−178.9 (6)	Ag1—P4—C55—C60	129.7 (6)
C7—C8—C9—C10	−0.2 (14)	C61—P4—C55—C56	−176.9 (6)
C8—C9—C10—C11	0.7 (14)	C67—P4—C55—C56	77.1 (6)
C9—C10—C11—C12	0.1 (14)	Ag1—P4—C55—C56	−51.3 (6)
C10—C11—C12—C7	−1.4 (13)	C60—C55—C56—C57	−0.5 (11)
C8—C7—C12—C11	2.0 (11)	P4—C55—C56—C57	−179.6 (6)
P1—C7—C12—C11	179.8 (6)	C55—C56—C57—C58	1.9 (13)
C1—P1—C13—C18	−99.4 (7)	C56—C57—C58—C59	−2.6 (14)
C7—P1—C13—C18	6.7 (8)	C57—C58—C59—C60	2.1 (15)
Ag1—P1—C13—C18	135.2 (6)	C56—C55—C60—C59	0.0 (13)
C1—P1—C13—C14	80.9 (6)	P4—C55—C60—C59	179.0 (7)
C7—P1—C13—C14	−173.1 (6)	C58—C59—C60—C55	−0.8 (14)
Ag1—P1—C13—C14	−44.5 (7)	C55—P4—C61—C62	92.7 (7)
C18—C13—C14—C15	0.8 (12)	C67—P4—C61—C62	−160.6 (6)
P1—C13—C14—C15	−179.5 (6)	Ag1—P4—C61—C62	−35.3 (7)
C13—C14—C15—C16	−0.1 (13)	C55—P4—C61—C66	−87.7 (7)
C14—C15—C16—C17	1.3 (14)	C67—P4—C61—C66	18.9 (7)
C15—C16—C17—C18	−3.1 (15)	Ag1—P4—C61—C66	144.2 (6)
C14—C13—C18—C17	−2.5 (12)	C66—C61—C62—C63	1.0 (12)
P1—C13—C18—C17	177.7 (7)	P4—C61—C62—C63	−179.4 (7)
C16—C17—C18—C13	3.7 (14)	C61—C62—C63—C64	−2.3 (14)
C25—P2—C19—C20	−96.7 (7)	C62—C63—C64—C65	2.6 (15)
C31—P2—C19—C20	9.0 (7)	C63—C64—C65—C66	−1.7 (15)
Ag1—P2—C19—C20	137.5 (6)	C64—C65—C66—C61	0.4 (14)
C25—P2—C19—C24	86.9 (6)	C62—C61—C66—C65	−0.1 (12)
C31—P2—C19—C24	−167.5 (6)	P4—C61—C66—C65	−179.6 (7)
Ag1—P2—C19—C24	−39.0 (6)	C61—P4—C67—C72	−86.6 (7)
C24—C19—C20—C21	−1.8 (11)	C55—P4—C67—C72	20.2 (7)
P2—C19—C20—C21	−178.3 (6)	Ag1—P4—C67—C72	149.7 (6)
C19—C20—C21—C22	2.1 (13)	C61—P4—C67—C68	89.4 (6)
C20—C21—C22—C23	−2.5 (13)	C55—P4—C67—C68	−163.8 (6)
C21—C22—C23—C24	2.7 (13)	Ag1—P4—C67—C68	−34.4 (6)
C22—C23—C24—C19	−2.4 (13)	C72—C67—C68—C69	2.1 (12)
C20—C19—C24—C23	1.9 (11)	P4—C67—C68—C69	−174.0 (6)
P2—C19—C24—C23	178.7 (6)	C67—C68—C69—C70	−2.3 (13)
C19—P2—C25—C26	8.7 (7)	C68—C69—C70—C71	2.6 (14)
C31—P2—C25—C26	−97.3 (7)	C69—C70—C71—C72	−2.6 (14)
Ag1—P2—C25—C26	135.9 (6)	C68—C67—C72—C71	−2.2 (12)
C19—P2—C25—C30	−171.2 (6)	P4—C67—C72—C71	173.8 (6)
C31—P2—C25—C30	82.9 (6)	C70—C71—C72—C67	2.4 (13)
Ag1—P2—C25—C30	−43.9 (6)	O1—S1—C73—F3	56.8 (13)
C30—C25—C26—C27	−3.7 (13)	O3—S1—C73—F3	178.9 (10)
P2—C25—C26—C27	176.5 (7)	O2—S1—C73—F3	−63.6 (12)
C25—C26—C27—C28	3.8 (15)	O1—S1—C73—F4	−65.8 (11)
C26—C27—C28—C29	−2.6 (16)	O3—S1—C73—F4	56.4 (11)
C27—C28—C29—C30	1.4 (15)	O2—S1—C73—F4	173.9 (10)
C28—C29—C30—C25	−1.4 (13)	O1—S1—C73—F5	−179.3 (11)
C26—C25—C30—C29	2.5 (12)	O3—S1—C73—F5	−57.2 (12)
P2—C25—C30—C29	−177.6 (6)	O2—S1—C73—F5	60.3 (12)
C25—P2—C31—C36	−163.3 (6)		
